# Significance of family history of cholelithiasis in a Pakistani population: A single center, descriptive cross-sectional study

**DOI:** 10.1097/MD.0000000000038925

**Published:** 2024-07-12

**Authors:** Mansab Ali, Amir Usman, Javeria Usman, Muhammad Abid, Wafa Najeeb, Muhammad Imran, Nour Fakih

**Affiliations:** a Department of General Surgery, The University of Lahore Teaching Hospital, Lahore, Pakistan; b Department of Gynecology and Obstetrics, The University of Lahore Teaching Hospital, Lahore, Pakistan; c Faculty of Medicine, University College of Medicine and Dentistry, The University of Lahore, Lahore, Pakistan; d Department of Natural Sciences, Lebanese American University, Beirut, Lebanon.

**Keywords:** family history, gallstones, genetics, Pakistan

## Abstract

Linkage studies have indicated a potential genetic predisposition to cholelithiasis. This study aims to determine the frequency of positive family history of gallstone disease in patients presenting with gallstones in a Pakistani population. A descriptive, cross-sectional study was conducted at the surgical department of the University of Lahore Teaching Hospital from June 30, 2023 to August 30, 2023. A total of 102 radiologically confirmed cholelithiasis patients were enrolled. Out of 102 participants, 75.5% (n = 77) were females, with a mean age at presentation of 42.1 ± 12.1 years. The study found that 32.4% (n = 33) of participants had a single family member with gallstones, 3.9% (n = 4) had 2 family members affected, and 1% (n = 1) had 3 family members affected. The attributable risk of genetics from our study was 37.2%. Additionally, there was no significant association between positive family history and earlier onset of disease. A significant percentage of Pakistani population may have gallstone disease due to genetic factors.

## 1. Introduction

Cholelithiasis is a prevalent diagnosis in the general population, regardless of geographical area, affecting up to 10% to 15% of the general population. However, in certain ethnicities, this may be as high as 60%.^[1]^ In Pakistan, a large percentage of the population is affected by gallstones; with prevalence ranging from 9% to 60%. Eighty percent of the affected are women, and the average age of diagnosis ranges from 35 to 40 years.^[2,3]^

While most gallstones are asymptomatic, in some cases, gallstone disease can significantly impact an individual’s quality of life. Cholecystitis, biliary colic, gallstone pancreatitis, ileus, and fistulization are potential consequences that might arise when gallstones, which initially have no symptoms, advance to a symptomatic stage.^[4,5]^

Moreover, there are some classic accepted risk factors identified for gallstone disease development. These include obesity, diabetes mellitus, estrogen and pregnancy, hemolytic diseases, and cirrhosis. Advancing age, female gender, and inborn errors of metabolism also contribute to the formation of gallstones.^[6]^ However, a less notable risk factor is that of genetics or family history.

Research conducted on families, twins, and linkage has confirmed the existence of a genetic propensity. This may also have implications for the ethnic predisposition observed in epidemiological data. For the most part, multiple genes or complex genetic pleomorphisms along with environmental factors or triggers are most likely to contribute to a genetic predisposition of developing gallstone disease.^[7]^

There is scarce data on cholelithiasis and family history in the Pakistani population. Establishing a causal connection could assist in developing screening guidelines for individuals who are deemed to be at higher risk. Therefore, this study aims to investigate the presence of a causal relationship, given the high prevalence of gallstone disease among patients in Pakistan.

## 2. Methodology

The study conducted was a descriptive, cross-sectional study at the University of Lahore Teaching Hospital from June 30, 2023, until August 30, 2023. After obtaining ethical approval from the Ethical Review Committee of the University College of Medicine and Dentistry, The University of Lahore (ERC/124/23/12), radiologically confirmed cholelithiasis patients presented to the surgical outpatient department were included using simple convenience sampling regardless of their age, gender, body mass index (BMI), co-morbidities, and presentation of cholelithiasis. Data were collected on a predesigned questionnaire. Patients were questioned regarding their demographics and family history of gallstones. Patients were enrolled after obtaining informed consent. All data were kept secure, and no identifiers were used. All data were entered into SPSS V 25 and analyzed. A *P* value of <.05 was considered as statistically significant.

## 3. Results

Out of 102 participants, 75.5% (n = 77) were female, while 24.5% (n = 25) were male. The mean age at the presentation was 42.1 years (Standard Deviation = 12.1). Two percent (n = 2) presented with choledocholithiasis, 1% (n = 1) presented with acute cholecystitis, and the rest presented with either symptomatic or asymptomatic cholelithiasis alone.

The mean weight of the patients was 69.3 kg (SD = 13.1). Among the patients, 5.9% (n = 6) had diabetes mellitus, 4.9% (n = 5) had both diabetes and hypertension, and 18.6% (n = 19) were hypertensive. Additionally, 1% (n = 1) of the patients were smokers.

Patients were assessed for a family history of cholelithiasis. Of the participants,62.7% (n = 64) did not have a family history of cholelithiasis. 32.4% (n = 33) had a single family member who had gallstones, 3.9% (n = 4) had 2 family members with gallstones and 1% (n = 1) had 3 family members affected by gallstones. Thus, the attributable risk of genetics from our study was 37.2%.

Risk factors were explored and 54.9% (n = 56) of women developed gallstones after a pregnancy or during a pregnancy. 38.2% (n = 39) of participants indicated that they had a diet rich in fatty meals. 15.7% (n = 16) of participants had extreme weight loss in a short period of time. 6.9% (n = 7) of participants indicated they had needed repeated transfusions at some point in their life. No patients were identified with endocrinopathies or congenital disorders that could lead to cholelithiasis.

A Chi-square analysis was done to see if there was an association between family history and earlier age onset of cholelithiasis. 21% (n = 8) patients aged 30 or younger had a positive family history while 18.8% (n = 12) did not; 79% (n = 30) aged 31 years or older had a positive family history (*P* = .77) while 81.3% (n = 52) did not show there was no significant association with positive family history and the earlier onset of disease. The odds of being younger and having gallstones with a positive family history are 0.86.

There was no significant association with earlier onset of disease at age <30 years and history of fatty meal intake (*P* = .825). There was no association with earlier onset of disease at age <30 years and pregnancy (*P* = .623).

## 4. Discussion

Pakistan is a country with a high burden of disease regarding gallstones, and most people affected are between the ages of 35 and 50 years old.^[2,3]^ Despite the existence of established risk factors such as consumption of fatty meals or pregnancy, the age distribution of individuals affected by gallstones remained in line with prior research, indicating a complex interaction between hereditary and environmental influences in the development of gallstones.

As prior evidence has shown that genetic predisposition may cause a higher incidence and odds of developing gallstones,^[7]^ our study indicated that 37.3% of patients had a positive family history, with up to 5% having more than 1 family member affected by gallstones. This finding raises the possibility that up to one-third of cases of gallstones in Pakistan may be linked to inheritability. However, it is crucial to understand that a variety of factors, including genetic predisposition, lifestyle choices, and environmental factors can affect gallstone disease.

Notably, a large population-based study in China found that a family history of gallstones increases the risk of developing stones by nearly 2.8 times that of an individual without a positive family history.^[8]^ Another study found that female sex (RR = 8.8) and a positive family history of cholecystectomy (Risk Ratio = 2.2) were factors in developing gallstone disease.^[9]^ This is comparable to our results, where we found that the majority of participants were females, and a large percentage had a positive family history. Interestingly, Nakeeb et al’s^[9]^ study also found that 29% of gallstone disease can be attributed to hereditary factors, which is similar to our 37.3%. Moreover, India is a country with a similar genetic make-up as Pakistan’s population, with similar environment and sociocultural factors; a study in India found that up to 69% of people had a positive family history when presenting with gallstone disease. This number is nearly double that of what we found in our study, which leaves a high level of disparity in the literature, evidencing the need for larger population-based studies in the subcontinent area.^[10]^ Another study compared the prevalence of a positive family history in patients with and without gallstone disease. They found that patients with gallstones had a positive family history in 28.6% of the cases, which is similar to our numbers, while controls without gallstones had a positive family history in 12.4% of cases.^[11]^This again shows that gallstone disease may have a heritable predisposition that needs exploration.

Moreover, a genetic variant recently has been identified (p.D19H) of the hepato-canalicular cholesterol transporter *ABCG5/ABG8* as genetic risk factor for gallstones. This gene can explain up to 10% of the gallstone risk.^[12,13]^ Genetic profiling, along with population-based screening to assess both the burden of gallstone disease and the family history positivity may help in delineating patients at risk of developing gallstones. Risk stratification can be done based on genetic and local profiling such as in the Northern Indian study, where the family history was positive in 69% of cases. This risk stratification may allow for better screening guidelines for the local populations and allow for choices such as prophylactic cholecystectomy.

Our study shows that nearly one-third of patients with gallstones may have a family history; however, the age of onset remains above 40 years for the majority of patients. It is possible that screening recommendations can be made for individuals in Pakistan with a positive family history above the age of 40; however, larger population-based studies are required to ascertain the risk of gallstone disease being genetically conferred in the local population. Our study offers a pivot with evidence that more work in this direction may decrease morbidity in patients with a positive family history where gallstones may be screened for and detected earlier before the development of complications and avoid difficult laparoscopic cholecystectomies.

Despite the potential of genetic screening to enhance risk stratification and guide clinical decision-making, several limitations warrant consideration. Our study’s cross-sectional design limits causal inference, and the relatively modest sample size may influence the generalizability of findings. Furthermore, the intricate interaction between genetic and environmental factors requires extensive longitudinal investigations in order to substantiate our findings and enhance the accuracy of risk prediction models.

## 5. Conclusion

Our study underscores the importance of considering genetic factors in the development of gallstone disease, particularly in high-prevalence regions such as Pakistan. Screening individuals with a positive family history may facilitate early detection and preventive measures, ultimately reducing morbidity associated with gallstones. We recommend further research to elucidate the genetic mechanisms underlying gallstone disease in the Pakistani population. Clinicians should consider family history when assessing risk and implementing screening strategies for gallstones.

## Acknowledgments

The authors thank all contributors who contributed valuable methods and data and made them public.

## Author contributions

**Conceptualization:** Mansab Ali.

**Investigation:** Mansab Ali, Amir Usman.

**Methodology:** Mansab Ali, Amir Usman, Javeria Usman.

**Supervision:** Mansab Ali.

**Writing—original draft:** Mansab Ali, Amir Usman, Javeria Usman, Wafa Najeeb.

**Validation:** Amir Usman, Javeria Usman, Muhammad Abid, Wafa Najeeb.

**Visualization:** Amir Usman.

**Resources:** Javeria Usman.

**Writing—review & editing:** Muhammad Abid, Muhammad Imran, Nour Fakih.

**Formal analysis:** Muhammad Imran.
